# Supplementary material for: Myths and Facts about Food Intolerance: A Narrative Review
Source: Nutrients. 2023 Nov 30;15(23):4969. doi: 10.3390/nu15234969 (PMC10708184; doi:10.3390/nu15234969)
Supplement: Supplementary file 1 [file nutrients-15-04969-s001.zip › nutrients-2723761-supplementary.pdf]

Supplementary Materials:

Table S1: Methods of diagnosis of lactose malabsorption and/or intolerance

**Test name**	**Method**
**H2 breath test and eventually CH4**	The lactose breath test is a commonly used, non-invasive diagnostic technique. During this test, a standard dose of 20-25 grams of lactose is ingested, and breath samples are collected at regular intervals, typically every 30 minutes for 3 hours (baseline, 90, 120, and 180 minutes). A positive indication of lactose intolerance is observed when the post-lactose breath hydrogen level increases by more than twenty parts per million (ppm) compared to the baseline measurement. Additionally, the presence of symptoms, as evaluated by a validated questionnaire, is taken into account to support the diagnosis of LI
**Lactose Tolerance Test**	The lactose tolerance test involves orally administering 50 g of lactose and monitoring blood glucose levels over 2 hours. Baseline blood glucose levels are measured before the test begins, and subsequent measurements are taken every 30 minutes. In individuals with normal lactose digestion, the ingested lactose is broken down into glucose, leading to an increase in blood glucose levels. Conversely, if there is little or no rise in blood glucose levels, it suggests inadequate lactose digestion, indicating lactose malabsorption. This test requires fasting and periodic blood sample collection, and it should only be conducted under the supervision of a healthcare professional in suitable clinical settings.<br><br>A shortened version of this test was proposed, lasting 30 minutes in total and taking only two measurements (baseline and 30-min).
**Duodenal lactase**	The measurement of duodenal lactase involves taking biopsies from the duodenum and examining them for lactase enzyme activity. This diagnostic approach is known for its high specificity, but it requires an invasive procedure. Typically, this method is reserved for ruling out secondary causes of lactose intolerance or when endoscopy is already being conducted for other purposes.<br><br>A Quick Lactose Intolerance Test use was described, which enables the Biopsy sample to be tested immediately and to obtain results in 20 minutes.
**Gaxilose test**	The gaxilose test is an innovative diagnostic tool used to assess the absorption of gaxilose, a non-metabolizable sugar, in the small intestine. If there is impaired absorption of gaxilose, it indicates reduced lactase activity, supporting the diagnosis of lactose malabsorption. Although the gaxilose test shows potential, it is still in the evaluation phase and is not widely available compared to other well-established testing methods.
**Genetic Test**	Genetic testing is a developing method that examines particular genetic variants linked to primary lactose malabsorption and its' use is suggested in Caucasians only. This test shows promising potential with increased sensitivity and specificity. However, its current availability is limited.

Table S2: Methods of diagnosis of histamine intolerance

Test name	Method
**Histamine challenge test**	The histamine challenge test could offer a diagnostic outcome and could help determine the minimum amount of ingested histamine that can induce symptoms in an individual. Nevertheless, there is no agreement on the precise threshold of histamine dosage that triggers symptoms exclusively in individuals with histamine intolerance. Some studies have employed a 75 mg histamine dose for this purpose, but it has induced symptoms in healthy individuals in various other studies, raising concerns about the potential influence of the nocebo effect in these instances. Given the potential for severe side effects during the histamine challenge, extended monitoring by medical personnel is essential. Reese et al suggest performing a titrated provocation with ascending doses of histamine hydrochloride at 2-hour intervals to determine the individually tolerated dose after the failure of the diet challenge.<br>Not validated yet
**Three-phase diet challenge**	In 2021, Reese et al. introduced the three-phase diet challenge as both a diagnostic approach and a therapeutic strategy to streamline the management of these individuals. However, before embarking on this diet challenge, it is essential to rule out IgE-mediated food allergies, mastocytosis, and the use of medications that could potentially affect histamine metabolism and distribution. Confirmation of the diagnosis requires the presence of at least two clinical manifestations, along with improvement observed after adhering to a low-histamine diet. The first phase consists of the restriction phase; phase two requires reintroducing different foods, taking into account the patient's symptoms and preferences; the third phase is a long-term diet. The specific duration of the low-histamine diet to confirm the diagnosis is not specified, but some research indicates a period ranging from 4 to 8 weeks. Not validated yet
**Histamine 50-skin-prick test**	In histamine intolerance diagnostics, a modified skin prick test variant is used. This delayed resolution of redness at the puncture site is observed in symptomatic patients compared to controls, indicating a potentially reduced ability of the body to degrade histamine administered intracutaneously. However, it is essential to note that this test may not necessarily reflect the histamine degradation in the small intestine, limiting its diagnostic accuracy. It has not been validated yet.
**DAO plasma levels**	Serum DAO levels can be measured through a blood test. Lower-than-normal levels of DAO may indicate a deficiency in the enzyme, which can contribute to histamine intolerance. It has not been validated yet.
**DAO enzymatic activity in plasma**	This test measures the activity of the DAO enzyme in plasma and entails a wide range of different methods, some measuring the degradation of histamine in blood samples over a specific period using enzyme-linked immunosorbent assay (ELISA) or radioimmunoassay (RIA), while others measuring the generation of by-products of this enzymatic reaction often using putrescine or cadaverine as a substrate.
**DAO enzymatic activity in colonic or small bowel biopsies**	Determination of DAO activity in the intestinal mucosa requires endoscopy to be performed which is not justified for this measurement per se. The results may be obtained through various methods. Not validated yet
**DAO genetic testing**	Genetic testing involves examining an individual's DNA extracted from blood or oral mucosa samples to identify particular Single Nucleotide Polymorphisms (SNPs) within the AOC1 gene. Currently, researchers have identified four distinct SNPs in the AOC1 gene that are associated with an elevated susceptibility to Histamine Intolerance. Not validated yet

**Urinary levels of histamine and its methylated metabolite, methylhistamine**	This test implies the determination of histamine and its metabolite 1-methylhistamine from urine by ultra-high performance liquid chromatography and fluorimetry. Not validated yet
**Histamine plasma levels**	Histamine plasma levels may be dosed with different methods such as colorimetry, fluorescence, ELISA, and chromatography, which are expensive and inconvenient. Not validated yet
**Fecal histamine levels**	The determination of fecal histamine levels is based on the assumption that since the microbiota of the gastrointestinal tract can be a significant source of histamine the measurement in stools could be an indirect measure of this production. Not validated yet
